# De buurtsportcoach: sport, bewegen en gezondheid verbonden

**DOI:** 10.1007/s12508-021-00303-0

**Published:** 2021-07-09

**Authors:** Wikke van Stam, Ine Pulles

**Affiliations:** Mulier Instituut, Utrecht, Nederland

**Keywords:** buurtsportcoach, sport als middel, lokaal beleid, landelijk beleidsprogramma, Community sport coach, Sport as a means, Local sports policy, Dutch sports policy

## Abstract

Sinds 2008 kunnen gemeenten met cofinanciering van het Rijk buurtsportcoaches inzetten om inwoners te motiveren om aan sport- en beweegactiviteiten deel te nemen. Deze snel groeiende beroepsgroep brengt lokale verbindingen tussen verschillende sectoren tot stand en versterkt sportaanbieders. Daarbij is sprake van een sterke verbinding met het gezondheids- en preventiebeleid, en leveren de buurtsportcoaches een belangrijke bijdrage aan de uitvoering van het Nationaal Sportakkoord en het Nationaal Preventieakkoord. Bijna alle gemeenten doen mee (99 % in 2021) en er zijn inmiddels gezamenlijk bijna 3.500 fte (6.000 personen) gerealiseerd (peildatum 1 september 2020). Lokale verschillen in de doelen en uitvoering van de werkzaamheden zijn groot, maar de buurtsportcoaches hebben gemeen dat ze zich enthousiast en daadkrachtig inzetten om maximaal resultaat te bereiken.

## Abstract (en)

Since 2008, the national government has provided municipalities with financial support to employ community sport coaches, who motivate inhabitants to participate in sports and physical activity. This rapidly growing group of professionals connects various sectors and sports providers at the local level. Their work is strongly linked to health and prevention policy. Almost all municipalities employ community sport coaches (99% in 2021) and together they employ almost 3,500 FTEs (6,000 persons). Despite large differences in their goals and activities at the local level, their enthusiasm and decisiveness in efforts to achieve maximum results are unifying characteristics among community sport coaches.

## Inleiding

In de loop der jaren zijn de opeenvolgende Rijksregelingen waarmee buurtsportcoaches werden aangesteld (Impuls Brede Scholen, Sport en Cultuur, Brede Impuls Combinatiefuncties, Brede Regeling Combinatiefuncties) telkens aangepast aan het op dat moment heersende sport- en politieke klimaat, en de uitkomsten van evaluaties. Het beleid van het kabinet was dat sporten en bewegen moesten bijdragen aan een gezondere samenleving en gezondere burgers, en de maatschappelijke samenhang moest bevorderen. Daarmee werden sporten en bewegen dus ingezet als middel om doelen van het volksgezondheidsbeleid (bewegen) en het welzijnsbeleid (meedoen) te halen. Naast en als opvolging van de regelingen die toen al bestonden, kwam in 2008 een impulsregeling waarmee gemeenten combinatiefunctionarissen (zo heetten ze destijds) konden inzetten om onderwijs, sport/bewegen en cultuur te verbinden. De focus op jeugd werd in 2012 verbreed naar sporten en bewegen (en cultuurdeelname) in de buurt voor alle Nederlanders, ongeacht leeftijd. Zo werden specifieke doelgroepen, zoals mensen met een beperking, steeds belangrijker. Ook was er een verbreding van sectoren. Waar sporten en bewegen voorheen verbonden werden met onderwijs (en cultuur), werden ze in 2012 uitgebreid naar andere sectoren, zoals zorg, welzijn, kinderopvang en het bedrijfsleven. Met de invoering van het Nationaal Sportakkoord in 2018, waarin buurtsportcoaches een belangrijk aandeel hebben gekregen, is nog meer verbreding te zien naar ‘inclusief sporten en bewegen’. Daarbij moeten belemmeringen om te sporten en bewegen voor *iedereen* worden weggenomen. Om het duurzame karakter van de inzet van buurtsportcoaches te benadrukken, heeft het Rijk in 2019 de naam van de impulsregeling veranderd naar Brede Regeling Combinatiefuncties. Daarbij is afgesproken dat de rijksgelden voortaan niet langer voor één jaar, maar voor vier jaar worden toegekend. Dit geeft gemeenten meer zekerheid voor de toekomst, waardoor plannen voor de toekomst en de daarbij behorende cofinanciering gemaakt en gerealiseerd kunnen worden.

Hoewel de verbinding van cultuur met andere domeinen nog steeds onderdeel is van de huidige regeling, ligt de focus van dit artikel op sport en bewegen. Daarbij zullen de ontwikkelingen en opbrengsten van de groeiende beroepsgroep van buurtsportcoaches centraal staan. We kijken daarbij specifiek naar het werk dat zij verrichten om Nederlanders gezonder te maken.

## Doelstellingen

Op landelijk niveau zijn in de bestuurlijke afspraken van de Brede Regeling Combinatiefuncties doelstellingen geformuleerd die het uitgangspunt vormen van de inzet van buurtsportcoaches [1]. Een aantal van deze landelijke doelstellingen heeft betrekking op het bevorderen van een gezonde leefstijl of het stimuleren van samenwerking met de gezondheidssector. Uit de jaarlijkse monitor onder deelnemende gemeenten blijkt dat het merendeel van de gemeenten lokale verbindingen tussen verschillende domeinen tot stand wil brengen en uitbouwen, motorische vaardigheden van de jeugd wil verbeteren en de ontwikkeling van kwetsbare jongeren met een verminderde actieve leefstijl wil stimuleren [2]. Hieronder een opsomming van alle doelen uit de landelijke regeling met daarbij het percentage van de deelnemende gemeenten dat buurtsportcoaches daarop inzet (deze doelen beslaan de hele Brede Regeling Combinatiefuncties, die voor 83 % uit sport/bewegen en 17 % uit cultuur bestaat [2]; hier is geen scheiding tussen sport/bewegen en kunst/cultuur mogelijk):

- sport-, beweeg- en cultuuronderwijs op en rond scholen versterken: 93 %;
- lokale verbindingen tot stand brengen en uitbouwen tussen gemeentelijke beleidsdomeinen en -voorzieningen, en tussen organisaties uit de sectoren sport, cultuur, onderwijs: 93 %;
- een leven lang inclusief sporten, bewegen en beoefenen van culturele activiteiten mogelijk maken: 85 %;
- verbeteren van motorische vaardigheden van jeugd en jongeren: 85 %;
- inzetten op groepen mensen die belemmeringen ervaren bij het (on)georganiseerd sporten en bewegen, en beoefenen van culturele activiteiten: 81 %;
- stimuleren van (talent)ontwikkeling van jeugd en jongeren: 75 %;
- duurzaam versterken en innoveren van sport-, beweeg- en cultuuraanbieders/vrijwilligersorganisaties, waaronder het vormgeven van een pedagogisch sportklimaat: 72 %;
- (talent)ontwikkeling van kwetsbare, minder kansrijke jongeren die (een risico op) een verminderd actieve leefstijl hebben: 72 %.
- bereiken en begeleiden van personen die in armoede leven, zodat zij ondanks hun financiële situatie kunnen sporten/bewegen en meedoen aan culturele activiteiten: 71 %;

Ook binnen het Nationaal Sportakkoord worden doelen gesteld waaraan buurtsportcoaches bijdragen. Negen op de tien buurtsportcoaches (91 %) leveren met hun werkzaamheden een bijdrage aan het streven dat iedere Nederlander een leven lang plezier aan sport en bewegen kan beleven [3]. De activiteiten van de buurtsportcoaches richten zich daarbij vooral op sportstimulering van inactieve of kwetsbare groepen. Ongeveer acht op de tien buurtsportcoaches zetten zich ervoor in om meer kinderen aan de beweegnorm te laten voldoen (78 %) en de motorische vaardigheden van kinderen toe te laten nemen (77 %).

## Opbrengsten

Omdat de landelijke regeling breed is ingestoken, verschillen de opbrengsten lokaal sterk. De ene gemeente zorgt voor veel extra sport- en beweeglessen voor kwetsbare groepen bij verenigingen, de andere verbetert de motorische vaardigheden van jonge kinderen en een derde probeert overgewicht tegen te gaan. Hierdoor is het lastig op landelijk niveau uitspraken te doen over de opbrengsten van de regeling. Overtuigend bewijs voor de relatie tussen de inzet van de buurtsportcoach in een wijk of gemeente en de sport- en beweegdeelname van burgers in die wijk of gemeente is dan ook niet gevonden [4, 5]. Wel zijn betrokkenen door de jaren heen steeds tevreden geweest met de inzet van de buurtsportcoach [5]. Een beeld dat we nu nog altijd zien: gemeenten geven gemiddeld een 8,1 als rapportcijfer voor tevredenheid over de inzet van buurtsportcoaches (peiling panel gemeenteambtenaren 2020, Mulier Instituut/VSG).

**Figure 1:**
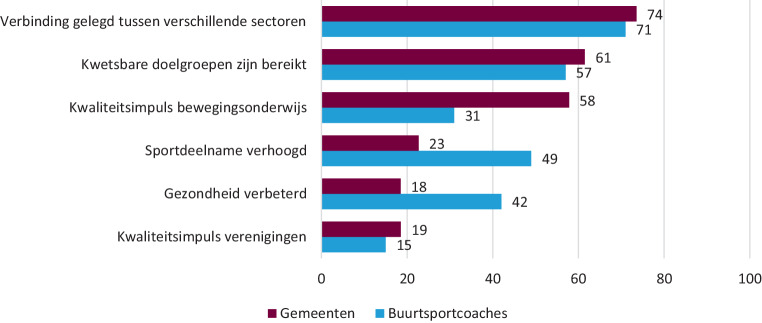

Alle betrokkenen (buurtsportcoaches en gemeenten) ervaren positieve resultaten van de inzet van buurtsportcoaches. In fig. 1 staan de resultaten die gemeenten en buurtsportcoaches het meest noemen.

Het tot stand brengen van lokale verbindingen tussen verschillende sectoren is een van de belangrijkste opbrengsten van de inzet van buurtsportcoaches (zie fig. 1). We zien nu nog steeds een sterke link met onderwijs (in 99 % van de gemeenten [2]), maar ook de welzijns- en zorgsector zijn inmiddels belangrijke sectoren voor de buurtsportcoach. Hieronder is te zien in hoeveel procent van de gemeenten sport en bewegen worden verbonden met organisaties uit de betreffende sector [2]:

- welzijn (bijvoorbeeld sociale wijkteams): 82 %;
- ouderen(zorg) (bijvoorbeeld ouderenwerk, woonvoorzieningen, verpleeghuis): 78 %;
- jeugd(zorg) (bijvoorbeeld instellingen, jeugd- en jongerenwerk): 76 %;
- gezondheidszorg (bijvoorbeeld huisartsen, fysiotherapeuten, ziekenhuizen, GGD, diëtisten): 76 %;
- gehandicaptenzorg (bijvoorbeeld woonzorginstellingen, dagbesteding): 58 %.

Ten opzichte van de beginsituatie twaalf jaar geleden zien we nu meer betrokkenheid vanuit de gezondheidssector, wat zich uit in zowel het leveren van de cofinanciering (lokale organisaties uit de welzijnssector betalen in 28 % van de gemeenten mee en lokale organisaties uit de zorg in 18 % van de gemeenten) [2], als het bepalen van de taken (die steeds meer gericht lijken te zijn op sport/bewegen als middel in plaats van sport als doel). In het Nationaal Preventieakkoord worden buurtsportcoaches genoemd als verbinder tussen de gezondheids- en sport/beweegsector, en samenwerkingspartner van zorg- en sportaanbieders bij het ontwikkelen van een passend aanbod dat ten goede komt aan de motorische vaardigheden van kinderen. Ook gezondheidsbevordering wordt gezien als een opbrengst van de inzet van buurtsportcoaches (fig. 1). De gemeenten en buurtsportcoaches die deze opbrengst zien, scharen deze onder verschillende pijlers van (positieve) gezondheid (peilingen panels gemeenteambtenaren 2020 (*n* = 69; Mulier Instituut/VSG) en buurtsportcoaches 2020 (*n* = 33; Mulier Instituut)):

- lichaamsfuncties (gezond en fit voelen): gemeenten 91 %, buurtsportcoaches 91 %;
- meedoen (goed contact hebben met andere mensen): gemeenten 85 %, buurtsportcoaches 88 %;
- mentaal welbevinden (vrolijk zijn): gemeenten 67 %, buurtsportcoaches 78 %;
- kwaliteit van leven (genieten van het leven): gemeenten 42 %, buurtsportcoaches 50 %;
- zingeving (vertrouwen hebben in de eigen toekomst): gemeenten 33 %, buurtsportcoaches 34 %;
- dagelijks leven (goed voor zichzelf zorgen): gemeenten 33 %, buurtsportcoaches 32 %.

Effectonderzoek naar de opbrengsten van de inzet van buurtsportcoaches op de verschillende doelen is op lokaal niveau erg lastig. Enerzijds komt dat doordat het ingewikkeld is om de effecten op het niveau van gedragsverandering bij mensen direct te koppelen aan de inzet van buurtsportcoaches. Omdat in een gemeente vele factoren invloed hebben op de sport- en beweegdeelname van inwoners, kan de directe relatie vaak niet vastgesteld worden. Anderzijds ontbreekt het vaak aan goede en structurele lokale monitoring. Bijna alle buurtsportcoaches houden wel iets bij van hun werkzaamheden. Maar tegelijkertijd heeft 78 % behoefte aan ondersteuning bij het inzichtelijk maken van de resultaten van hun werkzaamheden (van wie 33 % daar (heel) erg veel behoefte aan heeft; peiling panel buurtsportcoaches voorjaar 2019, Mulier Instituut).

## De beroepsgroep buurtsportcoaches

Buurtsportcoaches zijn de afgelopen jaren steeds meer ingezet, onder andere ter bevordering van de gezondheid. Waar in het eerste jaar 7 % van de gemeenten meedeed, is dat nu 99 %. In 2018 gaf een kleine helft van de gemeenten (43 %) aan meer fte te willen inzetten en iets minder dan de helft (45 %) niets aan het aantal fte buurtsportcoaches te willen veranderen; geen enkele gemeente wenste minder fte in te zetten (peiling panel gemeenteambtenaren 2018, Mulier Instituut/VSG). Gemeenten beschouwden de inzet van buurtsportcoaches als een duurzaam middel. Ze zijn tevreden over de buurtsportcoachinzet (peiling panel gemeenteambtenaren 2020, Mulier Instituut/VSG). De werkzame elementen van de regeling zijn in het bijzonder het structurele karakter van de cofinanciering van het Rijk en de vrijheid om de taken van buurtsportcoaches naar eigen lokaal inzicht in te vullen [6]. Inhoudelijk werkt de inzet van buurtsportcoaches volgens gemeenten vooral doordat ze sport en bewegen met andere sectoren verbinden, en door de mogelijkheid/stimulans tot lokaal maatwerk en de ‘doekracht’ die buurtsportcoaches in de lokale sportwereld brengen. De beroepsgroep die is ontstaan ziet zichzelf als verbindend (62 %), assertief/daadkrachtig (56 %) en enthousiast/gemotiveerd (39 %) [7].

We kunnen concluderen dat de afgelopen jaren een nieuwe beroepsgroep van professionals is ontstaan. De buurtsportcoaches zijn ingebed in het lokale beleid van zo goed als elke gemeente. Ze worden gewaardeerd, leggen connecties met vele sectoren en motiveren tot sport- en beweegdeelname. Ondanks grote lokale verschillen in de doelen en uitvoering van de werkzaamheden hebben buurtsportcoaches een aantal eigenschappen (verbindend, assertief/daadkrachtig, enthousiast/gemotiveerd) en een opdracht (Nederlanders motiveren voor deelname aan sport en bewegen) met elkaar gemeen. Ze beoefenen allen een specifieke functie op het snijvlak van beleid en praktijk, beschikken over lokale kennis en ontwikkelen op maat gemaakte activiteiten met behulp van eigen inzicht en ervaringen in de praktijk [5]. Dankzij die specifieke eigenschappen zullen de buurtsportcoaches als beroepsgroep in de toekomst in staat zijn om in te spelen op alle verschillende landelijke en lokale vragen die op ze af zullen komen. Dat zien we nu bijvoorbeeld ook bij alle maatregelen rond het coronavirus. De meeste reguliere sportbeoefening was aan banden gelegd, maar de beroepsgroep van buurtsportcoaches toonde veerkracht en sprong in op de veranderingen. Zo organiseerden buurtsportcoaches balkonlessen voor ouderen of mensen met een beperking en digitale beweeglessen voor kinderen [8]. Doordat de focus door dit virus nu vooral ligt op het gezond blijven van de bevolking en de preventie van ziekten, is het werk van de buurtsportcoach meer dan ooit van belang voor de gezondheidszorg.
